# Disposition of cimicoxib in plasma and milk of whelping bitches and in their puppies

**DOI:** 10.1186/s12917-015-0496-4

**Published:** 2015-07-31

**Authors:** M. Schneider, A. Kuchta, F. Dron, F. Woehrlé

**Affiliations:** Vétoquinol Global Development, 70200 Lure, France; Crosspatrick, Killala, Co. Mayo, Ireland

## Abstract

**Background:**

Caesarean section of bitches is a well recognized painful condition in dogs and it can be classified as a soft tissue surgery. Cimicoxib, a newly registered NSAID in European Union has a claim for the relief of pain in peri-operative conditions. However, in case of caesarean section, the main concerns of using NSAIDs are the transfer of the drugs into milk and its impact on the suckling pups. Thus, the aim of the present work was to evaluate the transfer of cimicoxib into the milk of 6 lactating bitches after a single oral administration of the drug on day 0 (just after whelping) and on day 28 at the target dose of 2 mg/kg. Another aim of the study was to evaluate the transfer of the drug from the milk into the suckling pups. Blood and milk samples were collected from the bitches after each administration on day 0 and day 28 and blood samples were drawn from the pups after suckling on day 28.

**Results:**

All bitches whelped without any complication and gave birth to 38 pups. After administration on D0, the mean observed plasma C_max_ in bitches was 0.5323 μg/mL and the mean area under the concentration-time curve extrapolated to the infinity, AUC_INF_, was 2.411 μg.h/mL. After administration on D28, only AUC_INF_ was significantly higher with a value of 3.747 μg.h/mL. In milk, after administration on D0, the mean observed C_max_ was 0.9974 μg/mL and the mean area under the concentration-time curve until the last measurable time point, AUC_last_, was 4.205 μg.h/mL. Out of 24 sampled pups on D28, only 2 animals had a sample with very low cimicoxib concentrations slightly above the limit of quantification (0.01 μg/mL).

**Conclusion:**

The presented data show that cimicoxib given by oral route to lactating bitches at a single dose of 2 mg/kg had a high transfer rate into the milk with a milk to plasma ratio of 1.7 to 1.9. The transfer rate to the suckling pups was low and no clinical abnormalities were detected in both bitches and pups.

## Background

Caesarean section of bitches is a well recognized painful condition in dogs [1]. It can be classified as a soft tissue surgery like ovariohysterectomy. In order to relief pain in peri-operative conditions, NSAIDs proved to be much helpful. Several NSAIDs are approved for the treatment of peri-operative pain in dogs and cimicoxib, a newly registered drug in European Union, has such a claim. However, in case of caesarean section, treatment of post-operative pain with NSAIDs is not a common practice. The main concerns of using NSAIDs are the transfer of the drugs into milk and its impact on the suckling pups [1]. At present, studies evaluating the transfer of NSAIDs into the milk of bitches are not published. The excretion of NSAIDs into milk was essentially studied in dairy cows in order to calculate withdrawal periods for a consumer protection purpose [2–5]. The excretion rates of these drugs into milk are generally quite low [2–5]. However, excretion of selective COX-2 inhibitors (coxib) in dairy milk is not described as these drugs are not yet approved in food animals. Coxibs being more lipophilic than classical NSAIDs [6], their excretion rate into milk might be higher.

**Fig. 1:**
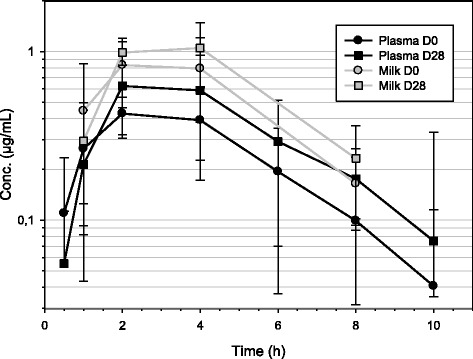
Mean concentration-time profiles of cimicoxib (semi-logarithmic scale) in plasma and milk of 6 bitches after a single oral tablet administration of a dose of 2 mg/kg (range 1.4 to 2.6 mg/kg) given just after whelping (D0) and 28 days later (D28). On D28, the milk concentration corresponds to the average out of 6 values for the time point of 1 h, out of 5 values for the time points of 2 and 4 h and out of 3 values for the time point of 8 h

In human medicine, NSAIDs are regularly used for the treatment of post-operative pain after elective or unscheduled caesarean section [7–11]. Use of these drugs is associated with faster recovery and lower requirements of opioid drugs [12–16]. The excretion of coxibs into human milk was studied with parecoxib [17]. The obtained milk concentrations of parecoxib and its active metabolite valdecoxib were quite low resulting in low exposure rates of the suckling children. The authors concluded that the administration of parecoxib to lactating women after cesarean delivery is unlikely to cause adverse effects in breastfed infants.

Thus, the main aim of the present study was to provide some information regarding the transfer of cimicoxib into the milk of bitches for the potential use of the drug in post-caesarean pain relief. The second aim of the study was to evaluate the transfer of the drug from the milk into the suckling pups.

## Results

All 6 bitches whelped without any complication and gave birth to 38 pups. The bitches were dosed successfully on study day 0 and 28 with no post-dosing vomiting. All bitches lost weight (range of weight loss was 0.5 – 3.8 kg) from study day 0 until study day 29. A decrease in bodyweight is not unexpected with lactating bitches. All pups were still alive on day 7 and they gained bodyweight from study day 7/8 to study day 29. On day 19, one pup was missing and was presumed to have been eaten by its mother. For all other pups, no abnormalities were detected during the scheduled veterinary examinations or clinical assessments. Cimicoxib was well tolerated in the bitches and in the pups throughout the study.

Mean concentration-time profiles of cimicoxib in plasma and in milk of the bitches are presented in Fig. 1. After administration on D0, the mean observed plasma C_max_ was 0.5323 μg/mL (sd = 0.08694 μg/mL) and the mean observed occurrence time of the C_max_, T_max_, was 2.83 h (sd = 1.33 h). The mean terminal half-life, T_½λz_, was 1.47 h (sd = 0.31 h). The mean area under the concentration-time curve extrapolated to the infinity, AUC_INF_, was 2.411 μg.h/mL (sd = 0.9771 μg.h/mL). The extrapolated part was low with a mean value of 3.14 % (sd = 1.41 %). The total clearance, Cl_F, was 0.8896 L/h.kg (sd = 0.2575 L/h.kg). The mean residence time extrapolated to the infinity, MRT_INF_, led to a mean value of 4.11 h (sd = 1.48 h). After administration on D28, the mean observed C_max_ was 0.6692 μg/mL (sd = 0.3088 μg/mL) which was not statistically higher than after administration on D0. The mean observed T_max_ was 2.67 h (sd = 1.03 h) which was close to the value obtained on D0 and not statistically different. The mean terminal half-life, T_½λz_, was 2.15 h (sd = 0.59 h) which was somewhat longer than after administration on D0, but the difference was not statistically significant. The mean AUC_INF_ was 3.747 μg.h/mL (sd = 1.821 μg.h/mL). The extrapolated part was still low with a mean value of 4.22 % (sd = 3.05 %). The AUC_INF_ value was statistically higher (*p* = 0.039) than the mean value obtained on D0. The total clearance, Cl_F, was 0.5867 L/h.kg (sd = 0.1472 L/h.kg) and was statistically lower (*p* = 0.031) than the clearance calculated on D0. The mean MRT_INF_ was 5.15 h (sd = 1.39 h).

In milk, after administration on D0, the mean observed C_max_ was 0.9974 μg/mL (sd = 0.250 μg/mL) which was about twice the value obtained in plasma on D0. The mean T_max_, was 3.17 h (sd = 1.33 h). The mean area under the concentration-time curve until the last measurable time point, AUC_last_, was 4.205 μg.h/mL (sd = 1.534 μg.h/mL). This value was also about twice the value obtained in plasma on D0. The mean residence time until the last measurable time point, MRT_last_ was 3.25 h (sd = 0.85 h). After administration on D28, milk samples could not be collected at every time point for all bitches because they produced much less milk than on D0. As the missing time points were located in the last elimination phase, only the observed C_max_ and the T_max_ could be obtained in the milk samples. The mean observed C_max_ was 1.146 μg/mL (sd = 0.2683 μg/mL) and the mean observed T_max_ was 3.00 h (sd = 1.10 h) which was close to the value obtained on D0.

For the pups, the time lapse between suckling and the first blood collection time point (1.5 h post bitch dosing) ranged from 20 to 29 min. Out of 24 sampled pups, only 2 animals had a plasma sample with very low cimicoxib concentrations, slightly above the limit of quantification. Both samples were taken 8.5 and 24 h after dosing the bitch. In the plasma samples of all the other puppies, the concentration of cimicoxib remained below the limit of quantification. Thus, although cimicoxib reached high concentrations in the milk of the bitches, the amount taken and/or absorbed by the pups is quite low.

## Discussion

To the best of our knowledge, this is the first report on excretion of NSAIDs into dog milk and on subsequent concentrations reached in suckling pups. Pharmacokinetics of cimicoxib were also described in other monogastric species: horses [18] and donkeys [19]. Compared to published pharmacokinetic data in dogs [20], the bitches in the present study could seem extensive metabolising animals. Indeed, the obtained terminal half-lives in plasma for days 0 and 28 were within a range of 1.09 to 2.97 h, whereas the mean terminal half-life in “poor metabolizers” was 8.0 ± 0.6 h [20]. The obtained mean terminal half-live on D0 (1.47 h) was even somewhat lower than the published value (2.9 h). The mean value obtained on D28 (2.15 h) was closer to the published value. Terminal half-life is a hybrid parameter depending both on volumes of distribution and on total body clearance. Lactation is known to modify both parameters with an increase in volume of distribution and/or an increase in clearance [21]. However, the most often encountered situation is an increase in total body clearance during early lactation [22] and hence a decrease in terminal half-life of drugs. This latter situation seems to be the case in our study on D0 because the mean clearance obtained, 0.8896 L/h.kg, is about two times higher than the clearance of non lactating dogs, 0.4959 L/h.kg [23]. At a later lactation stage on D28, the terminal half-life was increased almost significantly (*p* = 0.067) while AUC_INF_ values were also significantly increased (*p* = 0.039). This increase of exposure to cimicoxib may be explained only by a decrease of the total body clearance confirming that the shorter terminal half-life at early lactation was the result of an increased clearance on D0. The C_max_ and T_max_ values obtained in whelping bitches in the present study on D0 were close to published values in “normal” beagle dogs [23]. However, terminal half-life and AUC_INF_ values were lower in the present study which reinforces the clearance hypothesis.

The measured concentrations of cimicoxib in the bitch milk are higher than the concentrations measured in plasma leading to quite high milk to plasma concentration ratios of 1.7 to 1.9. Thus, cimicoxib seemed to be concentrated in the milk contrary to what was observed for parecoxib in human medicine [17]. This behaviour difference between the two drugs may be explained by the lipophilic nature of the drugs and by the milk composition. Dog milk contains about 10 % fat [24] whereas human milk contains about 3 to 5 % fat [25, 26]. The higher fat content of dog milk may increase the transfer rate of lipophilic drugs relative to human milk. Therefore, it might explain why the obtained milk to plasma concentrations ratio of cimicoxib in dog milk was higher than the ratio obtained with parecoxib in human milk. Concerning parecoxib, the volume of distribution of the drug in the dog was about 0.09 L/kg [27] which reflects a distribution essentially within body water. Its main active metabolite, valdecoxib, has a volume of distribution of about 0.9 L/kg [28] which is 10 times higher than its prodrug but still lower than the volume of distribution of cimicoxib which is about 1.1 L/kg [20]. As the volume of distribution of parecoxib is lower than the volume of distribution of cimicoxib, its transfer rate into the milk should be lower [21].

Despite these high milk to plasma concentration ratios, the intake of cimicoxib by the puppies seems to be very low. Indeed, cimicoxib concentration in the pups on D28 was below or slightly above the lower limit of quantification (0.01 μg/mL). In order to evaluate the dose of cimicoxib taken by the pups, a worst case scenario was considered. The C_max_ of cimicoxib in milk on D28 (1.146 μg/mL) was taken together with the average milk intake of the puppies. In the present study, this value was not measured; however, Oftedal [24] evaluated a mean milk intake of 175 g in 26 days old beagle puppies. Thus, as age of the puppies was very close to the age of the puppies in our study (28 days), this intake value was taken as a basis for our evaluation. Subsequently, the mean intake was calculated to be 201 μg of cimicoxib, assuming that the density of dog milk is close to 1. The mean weight of the pups in our study was 1.11 kg (sd = 0.23 kg) which is close to the weight of the pups in the manuscript of Oftedal [24]. The maximal cimicoxib dose taken by the pups was 0.18 mg/kg which is about 10 times lower than the dose administered to the bitches. Pups were not weighed on D0 in our study and therefore, estimation of the dose of cimicoxib taken after suckling is not straightforward. However, based on literature data [24, 29] a weight of 0.239 kg and a mean milk intake of about 100 g may be considered. Taking into account the C_max_ of cimicoxib in milk on D0, 0.9974 μg/mL, a maximal dose of about 0.42 mg/kg can be estimated. This dose is higher than the dose taken on D28, but it remained quite low.

## Conclusion

The presented data show that cimicoxib given by oral route to lactating bitches at a dose of 2 mg/kg had a high transfer rate into the milk with a milk to plasma ratio of 1.7 to 1.9. Twenty eight days old puppies suckling the milk from the dams treated with this single 2 mg/kg oral dose had plasma cimicoxib concentrations below or very close to the limit of quantification (0.01 μg/mL). Thus, the data obtained in the present work suggests that after administration of a single clinical dose of cimicoxib to whelping bitches, suckling puppies should be minimally exposed to the drug through the dam’s milk and no serious adverse effect should occur.

## Methods

### Animals and housing

Cimicoxib was given to 6 bitches shortly after whelping (D0) and the transfer of the drug into the milk of the dams was evaluated. The treatment was repeated about one month after whelping (D28) and the disposition of cimicoxib was evaluated again in the bitches as well as in their pups after suckling. The general study design is described in Table 1 and was approved by the Charles River Laboratories Preclinical Services Ireland Ethical Committee which has the authorization number AE19108.

**Table 1:** Experimental design

	Study days					
	D0	D1	D7	D27	D28	D29
Body weight	*bitches*	*-*	*-*	*-*	*bitches, pups*	*-*
Clinical assessment	*bitches*	*-*	*pups*	*-*	*bitches*	*-*
Drug administration	*bitches*	*-*	*-*	*-*	*bitches*	*-*
Blood sampling	*bitches*	*bitches*	*-*	*pups*	*bitches, pups*	*bitches, pups*
Milk sampling	*bitches*	*bitches*	*-*	*-*	*bitches*	*bitches*

-: not applicable

Six Beagle bitches from the breeding colony of Charles River Laboratories Preclinical Services at a late pregnancy stage weighing 10.8 to 18.7 kg were used and they were about 5 to 8 years old. Each bitch was enrolled onto the study when she gave birth to pups. Bitches were housed individually in pens measuring 4.0 m x 1.1 m (l x w). Inside each pen, a resting area with rubber matting was provided for each bitch. The pen was divided into two sections by a division down the middle. The dividing section had a gate to allow the animal access to both halves of the pen. The gate was used to separate the bitch from her pups when necessary. In each pen there was a whelping box measuring approximately 1.1 m x 0.7 m (l x w). An infra-red lamp was added at the time of whelping to provide a supplementary source of heat. After whelping, each pen housed 1 bitch and her litter. Bitches were fed with about 350 g/day with a specific dry food (Gilpa Trinkets, Gilbertson & Page ltd, UK). Pups were fed by suckling the milk of their dam.

Bitches were administered 1 or ½ 30 mg Cimalgex® tablet leading to an actual dose range of 1.4 to 2.6 mg/kg with a target dose of 2 mg/kg. Cimalgex® tablets were manufactured by Vétoquinol SA. The bitches were dosed once on study day 0 about 1 h after birth of the last pup. The technician assisting the birth of puppies assessed if the bitch had finished whelping by manual palpation. Bitches were fed after dosing on day 0 or at most 5 h before dosing. Bitches were dosed again once on day 28 before the pups were suckling. The bitches were fed after dosing on day 28.

On day 0 and day 28, all bitches were carefully observed for adverse events. Clinical assessments were carried out by a trained technician/veterinarian. Clinical assessments were performed prior to dosing and at 1.5 h ± 15 min post-dosing. The following parameters were assessed: behaviour, respiration, salivation/vomit, nervous signs, locomotion/musculature and faeces. When faeces were present, they were removed from the pen after each assessment. In addition, general health observations were carried out from day 0 to day 29.

On day 7 (±24 h), all pups that had survived, were microchipped, weighed and examined by a veterinarian. The pups from each litter that presented any evidence of illness at the veterinary examination were not qualified for participation. All the other pups which were considered as healthy were enrolled onto the study on day 7 (±24 h). General health observations were carried out throughout the study from day 7 to day 29.

### Sampling

Blood samples of 3 mL were collected post dosing (on days 0–1 and 28–29) from the jugular vein of the bitches in tubes containing lithium heparin on the following time points: 0.5, 1, 2, 4, 6, 8, 10 and 24 h. A sample was also taken pre-treatment before whelping. Blood samples of 1 mL were collected (on days 28 and 29) from the jugular vein of the pups on the following time points: 1.5, 4.5, 8.5 and 24 h after dosing the bitches or 0.5, 3.5, 7.5 h after suckling on day 28 and 0.5 h after suckling on day 29. A sample was also taken before dosing the bitch on day 27. Blood samples were collected from the pups that were confirmed to be suckling milk about 30 min before the first blood collection time point. A total of 4 pups per bitch (*n* = 24) was sampled. The tubes were centrifuged at 3500 rpm for 10 min at 4 °C. The resultant plasma collected from bitches was separated, transferred into 3 uniquely labelled clear polypropylene tubes (at least 500 μL of plasma per aliquot), and placed in a −80 °C deep freezer. There was only 1 plasma aliquot fraction for the pups.

Milk samples were collected manually from the mammary gland of the bitches into polypropylene tubes. To stimulate milk release, each bitch’s nipples were gently stripped by repetitive stroking motions. Each bitch’s mammary glands were either massaged or warmed with a warm cloth. About 1.1 mL of milk was collected on the following time points post dosing: 1, 2, 4, 8 and 24 h. On study days 0 and 1, a sample was taken from each animal at each time point. On study days 28 and 29, some samples could not be taken because there was not enough milk available. On study day 28, the first milk sample was taken just prior first suckling within 1 to 4 min. After collection, samples were mixed gently and separated, equally transferred into 2 uniquely labelled clear polypropylene tubes (at least 500 μL of milk per aliquot), and placed in a −80 °C deep freezer.

### Sample analysis

The concentration of cimicoxib in the plasma samples was determined according to a validated HPLC method described in Jeunesse et al. [20]. Briefly, cimicoxib was extracted by a solid liquid extraction process using HLB Oasis cartridges (Waters). Separation was achieved by a reverse phase column with an octadecylsilane stationary phase (Merck Lichrospher 100 RP18e (125x4) mm, 5 μm) using a guard column (Merck Lichrospher 100 RP18e (4x4) mm, 5 μm). UV detection was performed at 242 nm. Within-day and day-to-day coefficients of variation were less than 9 % and the accuracy ranged from 94 to 103 %. The limit of quantification of the method was 0.01 μg/mL. For the milk samples, the method was validated using cow milk as blank matrix. Only the extraction was altered using dichloromethane as an extracting solvent. The specificity of the method was tested against co-extracted milk impurities and against demethylated cimicoxib which is the major metabolite of cimicoxib [30]. The limit of quantification in the milk samples was 0.03 μg/mL. Within-day and day-to-day coefficients of variation were less than 7 % and the accuracy ranged from 96 to 105 %.

### Pharmacokinetic evaluation

The individual plasma data sets were submitted to a non compartmental analysis by means of the WinNonlin® software (version 5.0.1). Actual doses of cimicoxib (mg/kg) and actual blood sampling times were used for the analyses. The best-fitting slopes of the terminal phase, λz, were computed by log-linear regression using the best adjusted R-square with at least three time points (excluding the C_max_). Area Under the Curve (AUC) values were computed using the Linear Trapezoidal, Linear Interpolation rules. The main pharmacokinetic parameters were calculated. The following parameters obtained on D0 and on D28 were compared using paired t tests with a significance threshold of 5 %: C_max_, T_max_, T_½λz_, AUC_INF_ and Cl_F.

Only the milk data sets collected on D0 were submitted to a non compartmental analysis. There were not enough samples collected on D28 for performing a pharmacokinetic analysis. The same rules for non compartmental analysis (plasma samples) were applied to the milk samples. The last elimination slope, λz, was not calculated because there were not enough time points.

## Abbreviations

COX: Cyclo-oxygenase
HPLC: High performance liquid chromatography
NSAID: Non steroidal anti-inflammatory drug
Rpm: Round per minute
SD: Standard deviation
UK: United Kingdom
UV: Ultra-violet
